# Long Non-Coding RNAs (lncRNAs) in Cardiovascular Disease Complication of Type 2 Diabetes

**DOI:** 10.3390/diagnostics11010145

**Published:** 2021-01-19

**Authors:** Nurruzanna Ismail, Noraidatulakma Abdullah, Nor Azian Abdul Murad, Rahman Jamal, Siti Aishah Sulaiman

**Affiliations:** UKM Medical Molecular Biology Institute (UMBI), Universiti Kebangsaan Malaysia, Jalan Yaa’cob Latiff, Cheras, Kuala Lumpur 56000, Malaysia; nananinaismail@gmail.com (N.I.); noraidatulakma.abdullah@ppukm.ukm.edu.my (N.A.); nor_azian@ppukm.ukm.edu.my (N.A.A.M.); rahmanj@ppukm.ukm.edu.my (R.J.)

**Keywords:** long noncoding RNA, cardiovascular disease, type 2 diabetes, biomarker, diagnosis, insulin secretion

## Abstract

The discovery of non-coding RNAs (ncRNAs) has opened a new paradigm to use ncRNAs as biomarkers to detect disease progression. Long non-coding RNAs (lncRNA) have garnered the most attention due to their specific cell-origin and their existence in biological fluids. Type 2 diabetes patients will develop cardiovascular disease (CVD) complications, and CVD remains the top risk factor for mortality. Understanding the lncRNA roles in T2D and CVD conditions will allow the future use of lncRNAs to detect CVD complications before the symptoms appear. This review aimed to discuss the roles of lncRNAs in T2D and CVD conditions and their diagnostic potential as molecular biomarkers for CVD complications in T2D.

## 1. Introduction

Type 2 diabetes (T2D) is a disease of high blood glucose (hyperglycemia) due to insulin action failure. About 463 million individuals have diabetes worldwide, with 90% of them T2D patients [1]. The number is going to increase to 700 million affected people worldwide in 2045 [1]. The increasing prevalence of diabetes worldwide is a burden, as most T2D patients will develop macrovascular and microvascular complications, which lead to various severe health problems and mortality [2,3,4]. Despite advances and improvements in the treatment options for T2D, the majority of deaths in T2D patients are due to cardiovascular diseases (CVD) [5,6,7,8]. People with diabetes have a CVD mortality risk 3- to 4-fold higher than those without diabetes [6,9]. Both T2D and CVD share some pathophysiological pathways and risk factors, particularly in the development of vascular damage, causing atherosclerosis [2,4,10]. These pathophysiologic changes will be elaborated in the next section. Early identification of which diabetic patients will develop CVD is important to reduce the severe disease outcomes and mortality.

The mapping of the whole human genome and transcriptome showed that the protein-coding genes account for only 2% of the whole human genome, where the rest consist of undefined or non-coding regions [11,12]. Recent evidence showed that 98% of the genome are the functional non-coding RNAs [12,13,14,15]. Non-coding RNAs (ncRNAs) are grouped into their sizes: (1) the small ncRNAs (size <200 bp) such as microRNA (miRNA) and some of the circular RNA (circRNA), and (2) the large ncRNAs such as long non-coding RNA (lncRNA) [13,14,15]. Among these ncRNAs, miRNA is most studied and comprehensively reviewed in T2D and CVD development [16,17,18], as well as being potential therapeutic targets [19,20,21]. In comparison, research on lncRNA and circRNA in T2D or CVD is still limited [22,23,24,25]. Previous reviews have discussed the interactions of these ncRNAs (miRNA-lncRNA-circRNA) in contributing to CVD development [26,27,28] and diabetes-related diseases [29,30,31]. Since lncRNAs are stably present in the biological fluids [32], they have similar potential to miRNA as new biomarkers or therapeutic targets. This review aimed to summarize the recent findings of circulating lncRNAs as the potential biomarkers for CVD complications in T2D. How these lncRNAs are involved in CVD development in T2D will also be explored. 

## 2. Long Non-Coding RNAs (lncRNAs) in Type 2 Diabetes (T2D)

Long non-coding RNAs (lncRNAs) are the RNA molecules with a size of more than 200 nucleotides and without the protein translation capacity [33,34]. The biogenesis and functions of lncRNAs have been discussed extensively [33,34]. LncRNAs are heterogeneous due to their genomic origins, in which they arise from the unconserved regions of the genome [33,34]. They are similar to messenger RNAs in their primary structure, with some of the lncRNAs having a 3′polyadenylation tail, although this 3′tail is not necessarily for their functions [35]. For the lncRNAs’ secondary and tertiary structures, the structural motifs are relatively conserved and vital for their functions [36,37]. About 27,919 human lncRNAs have been reported, and 70% of them are functional [38]. LncRNAs regulate the transcription process by interacting with the proteins, RNA, and DNA molecules in the cis- and trans-regulation activities [33,34]. In the cis-regulation, lncRNAs act on the neighbouring genes on the same allele of their origin. In the trans-regulation, lncRNAs exert their regulatory activities on distant locations from their origin [33,34]. Among the reported regulatory functions, lncRNAs interact with the chromatin-modifying complexes to facilitate the chromatin remodeling and initiate the transcription of the downstream genes [33,34]. LncRNAs also act as scaffolds to stabilize complexes, allow protein–protein or protein–RNA formations, and direct these complexes to specific locations on the DNA for the intended regulation [33,34]. Another known function of lncRNA is to act as a microRNA (miRNA) sponge, in which lncRNA binds to the miRNA to prevent the target gene silencing [33,34]. Previous GWAS studies identified genetic variants in the lncRNA antisense non-coding RNA gene at the INK4 locus (*ANRIL*) that was associated with a higher risk of having T2D [39] and coronary artery disease (CAD) [40], therefore, suggesting the involvement of lncRNAs in diabetic CVD complication. First, we summarized and discussed the reported lncRNAs involved in the T2D environment (Table 1).

### 2.1. LncRNA in Insulin Secretion

**Figure 1:**
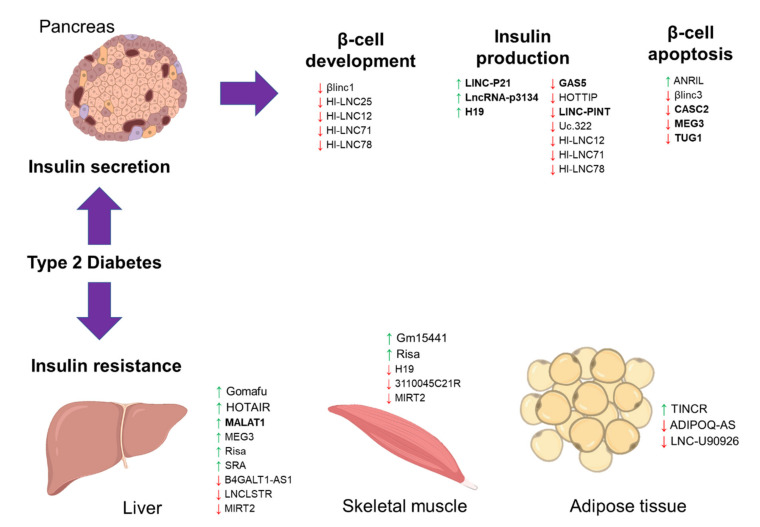
The schematic diagram illustrates the long noncoding RNA (lncRNA) involvement in Type 2 diabetes (T2D). The diagram shows the involvement of previously published long noncoding RNAs (lncRNAs) in T2D, focusing on insulin secretion and resistance. A green upward arrow indicates the upregulation of the lncRNA expression, a red downward arrow indicates the reduced expression, and the bold lncRNAs exist in the circulating biological samples for diagnostic potential.

Dysregulation of the lncRNAs involved in the pancreatic β-cell development and function could contribute to the failure of or reduced insulin secretion (Table 1 and Figure 1) [71]. One of the main events in T2D disease development is the failure of the pancreatic β-cell compensatory insulin secretion in response to insulin resistance [72]. The increase of the insulin secretion depends on the ability of each β-cells to produce more insulin (β-cell function per cell) or high numbers of existing β-cells in the islet (β-cell mass) [72]. Therefore, dysregulated lncRNAs in the islet and β-cells development will cause insulin secretion failure. One such lncRNA is the β-cell long intergenic non-coding RNA 1 (*βlinc1*), a conserved lncRNA located upstream of the NK2 homeobox 2 (*NKX2-2*) gene (an essential islet transcription factor) [73]. A knockout mouse model study revealed that lncRNA *βlinc1* is necessary for proper coordinated islet-specific transcription factors [44]. Deleting *βlinc1* in the mouse model causes defective islets and disrupts the glucose homeostasis in the adult knockout mice [44]. Transcriptomic profiling of human islets revealed that some lncRNAs are exclusively expressed in the islets [41]. One particular lncRNA is the human islet long non-coding RNA 25 (*HI-LNC25*) that is located near to MAF bZIP transcription factor B (*MAFB*) gene (an essential gene for islet maturation), and this lncRNA regulates the expression of GLIS family zinc finger 3 (*GLIS3*, islet transcription factor) [41]. In the same study [41], the expressions of lncRNA *KCNQ1* opposite strand/antisense transcript 1 (*KCNQ1OT1*) and *HI-LNC45* lncRNAs were upregulated and downregulated, respectively, in T2D patients. Although their functions are unknown, lncRNA *KCNQ1OT1* is one of the T2D susceptible loci [74]. Other reported islet-specific lncRNAs are the *HI-LNC12*, *HI-LNC78*, and *HI-LNC71*, in which the knockdown of these three lncRNAs caused impaired insulin secretion [49]. Importantly, lncRNA *HI-LNC71* is located upstream of the pancreatic and duodenal homeobox 1 (*PDX1*) gene in an antisense orientation, and a knockdown of lncRNA *HI-LNC71* in the mice reduced the expression of *PDX1*. Since PDX1 is the main regulator of pancreatic development and β-cell function, the loss of lncRNA *HI-LNC71* may indeed cause disruption in pancreatic function and subsequently induce T2D [49]. A more recent study also reported another lncRNA, *LINC00261*, that is also important for the islet differentiation, in which it interacts with the islet transcription factor, forkhead box A2 (*FOXA2*) [75]. These findings suggest that most of these lncRNAs are from the regions near the essential islet differentiation and development factors, indicating the regulatory role of these lncRNAs during the gene transcription process.

In terms of β-cell insulin production and secretion, lncRNA growth arrest-specific 5 (*GAS5*) is the most reported lncRNA in the association with T2D. In a study of the T2D mouse model, lncRNA *GAS5* expression was lower in the islets of T2D mice, and silencing of this lncRNA expression caused impaired insulin synthesis and secretion in the MIN6 cells [46]. A similar observation was seen in a study of T2D patients, in which the circulating expression of *GAS5* was reduced in T2D patients [47]. Silencing of *GAS5* expression in the rat islet cell line reduced the expression of multiple microRNAs (miR-29a-3p, miR-96-3p, and miR-208a-3p) and subsequently reduced these microRNAs’ targets expressions, including the insulin receptor, insulin receptor substrate, and phosphoinositide-3-kinase regulatory subunit-1 [47]. Transcriptomic profiling of the human islets also found *LOC283177* is co-expressed with the insulin synthesis and secretion regulators, including MAP-kinase activating death domain (*MADD*), synaptotagmin 11 (*SYT11*), and paired box 6 (*PAX6*) [76]. Besides that, lncRNA *uc.322* is also exclusively expressed in pancreatic β-cells [55]. Overexpression of lncRNA *uc.322* in the MIN6 cells caused greater glucose-stimulated insulin secretion via the upregulations of the *PDX1* and forkhead box O1 (*FOXO1*) expressions [55]. Another lncRNA is the long intergenic non-protein coding RNA, p53 induced transcript (*LINC-PINT*), that may regulate insulin synthesis and secretion, and this lncRNA expression was reduced in the T2D mouse model [51] and plasma of T2D patients [77]. In contrast, expression of *LINC-P21* was higher in the serum of T2D patients, and silencing of this lncRNA resulted in better glucose-stimulated insulin secretion of the INS-1 cells via miR-766-3p/*NR3C2* axis [42]. Recent profiling of T2D patients showed that serum exosomal *lncRNA-p3134* was enriched in T2D patients and correlated with fasting blood glucose and HOMA-β levels [43]. This enrichment of *lncRNA-p3134* expression was only seen in serum exosomes, not in the serum samples alone, indicating a specific enrichment in the extracellular vesicles. Also, *lncRNA-p3134* regulates insulin secretion by promoting the expressions of critical modulators (*PDX1*, *MAFA*, *GLUT2*, and *TCF7L2*), possibly via the PI3K/Akt/mTOR signaling in the pancreatic β cells [43]. 

The progressive loss of β-cells is common in T2D disease development [78]. LncRNA is also implicated in β-cell death. A previous study of human and mice islets showed that lncRNA *βlinc3* expression was low in T2D condition, and this lncRNA promotes β-cell apoptosis [45]. Another reported lncRNA is taurine up-regulated 1 (*TUG1*) that also plays a role in β-cells, and a reduced expression of TUG1 causes apoptosis and impaired insulin secretion [54]. Maternally expressed 3 (*MEG3*), a maternally imprinted lncRNA, was also reduced in the islets of T2D patients [52]. This reduced expression was due to hypermethylation on the cluster region of *MEG3* location, caused the suppression of the same cluster’s microRNAs [52]. Functional analysis of these dysregulated microRNAs showed that they are involved in β-cell apoptosis and T2D pathogenesis [52], which may imply that *MEG3* could also regulate β-cell function. Further investigation was done in the T2D mouse model, in which silencing of *MEG3* expression led to impaired insulin production and β-cell apoptosis [53]. Circulating lncRNA *H19* imprinted maternally expressed transcript (*H19*) was reduced in T2D patients, and this low expression of lncRNA *H19* was associated with the increase of miR-29a and miR29b expressions [48]. High expression of the miR-29 family causes β-cell death and dysfunction [79], thus may imply that lncRNA *H19* presence is important for normal β-cell function. This *H19* role is supported by another study of mice islets, in which high expression H19 promoted the expansion of β-cell mass in response to increasing glucose demand [80]. Another is the lncRNA *HOXA* distal transcript antisense RNA (*HOTTIP*), which was lower in the T2D mouse model, and silencing of this lncRNA caused reduced insulin secretion and its regulators expressions (*PDX1* and *MAFA*) [50]. In-depth investigation showed that lncRNA *HOTTIP* also regulates cyclin proteins and β-cell cycle [50]. These interactions between the lncRNA and microRNAs may explain how the loss of these lncRNAs could contribute to dysregulation of the insulin secretion vital regulators. 

### 2.2. LncRNA in Insulin Resistance

Insulin resistance is due to the impaired response of insulin-responsive cells, and lncRNA may regulate insulin signaling (Table 1 and Figure 1). One such lncRNA is *MEG3* that was higher in the liver of the high-fat-fed and obese mouse models [59]. High *MEG3* expression causes hepatic insulin resistance by increasing the expression of *FOXO1*, which then promotes hepatic gluconeogenesis. Notably, the increase of *MEG3* expression due to high-fat diets caused increased histone acetylation, removing the epigenetic suppression on the *MEG3* cluster [59]. Similarly, lncRNA metastasis-associated lung adenocarcinoma transcript 1 (*MALAT1*) was also increased in the obese mouse model’s liver and promoted insulin resistance [58]. *MALAT1* stabilizes the sterol regulatory element-binding transcription factor 1c (SREBP-1c) in the nucleus and causes excess lipid accumulation in the hepatocytes [58]. Another reported lncRNA contributing to hepatic lipid accumulation is the lncRNA steroid receptor RNA activator (*SRA*). LncRNA *SRA* suppresses adipose triglyceride lipase (*ATGL*) expression and inhibits fatty acid oxidation, causing hepatic lipid accumulation [61]. A protective liver lncRNA, liver-specific triglyceride regulator (*LNCLSTR*), was shown to positively regulate plasma triglyceride clearance via interacting with the TDP-43/FXR/APOC2 pathway [63], thus may be beneficial for treating insulin resistance. A recent review has discussed these lncRNAs in-depth and discussed their potential as early diagnostic markers for insulin resistance [81]. However, many of these lncRNAs are also present for other metabolic diseases such as obesity and fatty liver; thus, their roles are not specific to the T2D environment. 

### 2.3. Circulating lncRNAs as Biomarkers for T2D

Some of these lncRNAs are present in the circulating biological fluids, and thus some studies determined the potential of these lncRNAs as biomarkers for T2D (Table 2). One such study is the cohort of US army veterans, in which the serum lncRNA *GAS5* level was lower in the T2D patients, and those with serum *GAS5* level lower than 10 ng/µL have 12 times greater risk to have T2D [82]. Moreover, the diagnostic analysis using the Receiver operating characteristic (ROC) analysis showed that serum *GAS5* level could differentiate the T2D patients from the healthy controls with an area under the curve (AUC) of ROC of 0.81, with 67.3% specificity and 85.1% sensitivity [82]. A recent systematic review reported that out of the seven studies, circulating expression of lncRNAs could differentiate the individuals with T2D from the healthy controls with a minimal sensitivity (0.71) and specificity (0.66) [83]. A total of 11 blood lncRNAs were identified from the previous studies, in which three lncRNAs were upregulated (ENST00000550337.1, TCONS_00007244, and TCONS_00000886 [84]), and eight lncRNAs were downregulated (*VIM-AS1*, *CTBP1-AS2* [85], *LY86-AS1*, *HCG27_201* [86], *LINC00523*, *LINC00994* [87], *CASC2* [88], and *GAS5* [82,89]) in T2D individuals. In terms of genetic susceptibility of T2D, lncRNA *ANRIL* is consistently reported to increase the risk of having T2D and CAD [90]. In this locus, the genetic alterations are different for T2D and CAD, in which most of the T2D genetic risk variants are located distal to the last exon, except one variant (rs564398) that is associated with both CAD and T2D conditions [91]. Although the mechanism of how *ANRIL* regulates glucose metabolism and insulin secretion is unknown, the circulating expression of *ANRIL* was higher in T2D patients, patients with myocardial infarction (MI) only, and much higher in T2D patients with MI, thus indicating the role of *ANRIL* in CVD progression of T2D patients [92]. Furthermore, identification of the role of these T2D-lncRNAs may provide a better understanding of the molecular network in the development of T2D. As recently reported, the treatment of glucagon-like peptide-1 receptor agonists (GLP1RAs) in the T2D cell model showed that lncRNA NONRATT027738 is in the core of the molecular networks that resulted in the β-cell improvements following the GLP1ARs treatment [93]. 

## 3. Cardiovascular Disease (CVD) Complication in T2D

CVD encompasses the anomalies in the heart and vascular circulatory system, including myocardial ischemia/injury and infarction (MI), atherosclerosis, hypertension, myocarditis, valvular disease, coronary artery disease, and arrhythmias [2,4]. With other risk factors such as obesity, aging, and diabetes, these anomalies eventually lead to heart failure (HF) [2,4]. There is a significant gap in understanding the mechanisms and the signaling pathways involved in these CVD events in T2D. What is known is that the pathophysiology of the CVD complication in T2D depends on the balance between the damage or dysfunctional changes that occur due to the T2D environment and the response of the endogenous protective factors of the heart [2]. These changes due to T2D in the vascular system and blood cause hemodynamic stress on the heart [2]. As a response, the heart or endothelial cells make a series of genetic, molecular, and cellular changes known as the cardiac remodeling process, including cardiac hypertrophy, fibrosis, inflammation, and vascular changes [94]. Thus, depending on T2D individuals, their physiology’s functional changes at the microvascular level can differ from at the macrovascular level. Therefore, they may vary the implications with respect to future CVD risk and outcomes. 

In the T2D environment, one of the early critical events for CVD complications is the increase in arterial stiffness and endothelial cell damage that contributes to atherosclerosis [95,96]. The high levels of circulating glucose and lipids are significant determinants for both arterial stiffness and carotid intimal media thickness (IMT) [97,98], and this association is only evident in T2D patients [99]. Thus, further confirming that there are specific metabolic changes in the T2D environment in those with CVD complications. The elevated circulating glucose and triglycerides, particularly the low-density lipoprotein (LDL), are taken in by the arterial intima (a layer of the endothelial cells in the artery) and consequently, recruits the macrophages to the site (an early formation of the atherosclerotic plaque), to take up the lipoproteins and causes their differentiation into the foam cells [100]. 

Eventually, this event leads to high production of inflammatory cytokines and chemokines together with excessive release of the reactive oxygen species (ROS) and AGEs, thus activating the nuclear factor-κB (NFκB), activator protein-1 (AP-1), endothelin-1 (ET-1, a vasoconstrictive signal), pro-thrombotic tissue factors, and plasminogen activator inhibitor-1 (PAI-1) [100,101,102]. The increased levels of these molecules contribute to inflammation, vasoconstriction, and thrombosis [100,101,102]. Additionally, the presence of insulin resistance also contributes to greater ROS production [103]. ROS activates higher expressions of adhesion molecules in endothelial cells, which further recruits more leukocytes into the plaque and increases the formation of the extracellular matrix (ECM) in this area [100,103]. Similar to insulin resistance, disruption of protein kinase C action also contributes to impaired angiogenesis, vasodilation, and greater leukocyte recruitment to the plaque by inhibiting PI3K signal [100]. This atherosclerotic plaques block the blood flow and lead to life-threatening events such as myocardial infarction (MI) and stroke [100]. 

As a response to these changes within the T2D environment, cardiac remodeling, which is defined by genetic, molecular, cellular changes that result in changes in the shape, size, and function of the cardiac cells, will occur [94]. The failure of this remodeling can result in cardiac dysfunction or, in the worst cases, heart failure (HF) [104]. Although the exact mechanisms or effects of T2D on cardiac remodeling are unknown, evidence of either dilated or restrictive left ventricular (LV) phenotypes is seen in T2D patients [105]. One of the reported failed cardiac remodeling mechanisms is where the heart activates the fetal expression of cardiac genes that causes the loss of ventricular function [94]. This event is asymptomatic at the start but eventually will lead to HF [104]. Another mechanism is the ion channel remodeling, such as the inactivation of the sodium channels, changes in calcium and potassium channels resulting in ventricular arrhythmias [106]. These ion changes impair the cellular contractility and prolong the cardiac action potential (AP) due to the reduced membrane repolarizing current [106]. 

Another cause for HF is the reduced expression of Connexin 43 (*CX43*) [107]. *CX43* is the most highly expressed gap junction protein in the heart and is responsible for cell-to-cell communication for electrical and physical purposes [108]. A combination of the reduced *CX43* expression and sodium channel dysfunction causes a reduction in intercellular coupling current and membrane excitability [109], thus causing the prolongation of the QT interval and arrhythmias [94]. A prolonged QT interval can induce Torsade de Pointes of ventricular tachycardia or ventricular fibrillation, and sudden cardiac death [94,104]. Besides those factors, an increase of collagen content during fibrosis in the myocardium can disrupt the electrical conduction and arrhythmias [94]. The myocardium’s extracellular collagen matrix preserves the muscle fibers, cardiac cell alignment, and ventricular shape [110]. Cardiac remodeling to increase the collagen content causes the thickening of existing fibrillar collagen, stiff myocardium, and dysfunction in the left ventricular diastolic, a condition that is often seen in cardiac hypertrophy [110,111]. This reduced collagen content will also cause ventricular remodeling in HF [112]. Coronary blockage (atherosclerotic plaques) can also induce the breakdown of fibrillar collagen together with infarction (tissue death or necrosis) and loss of cardiac tissue integrity [94]. Thinning of these infarcted walls and ventricular dilation increases the risk of myocardial rupture, as seen in myocardial infarction (MI) patients [94]. 

## 4. Long Non-Coding RNAs in CVD

### 4.1. LncRNA in Atherosclerosis

**Figure 2:**
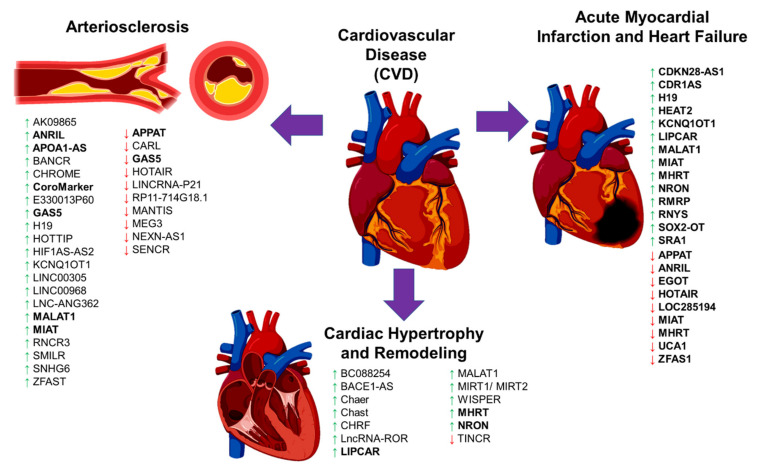
The schematic diagram illustrates the long noncoding RNA (lncRNA) involvement in cardiovascular disease (CVD) complications. The diagram shows the involvement of previously published long noncoding RNAs (lncRNAs) in cardiovascular disease (CVD), focusing on arteriosclerosis, cardiac remodeling/hypertrophy, myocardial infarction, and heart failure. A green upward arrow indicates the upregulation of the lncRNA expression, a red downward arrow indicates the reduced expression, and the bold lncRNAs exist in the circulating biological samples for diagnostic potential.

In atherogenesis plaque development, endothelial cell (EC) dysfunction, inflammatory leukocytes with the release of cytokines and chemokines cause phenotypic changes in the vascular smooth muscle cells (VSMC) from a contractile-quiescent state to an activated proliferative state and migration with the subsequent formation of the extracellular matrix or the plaque [100,101]. EC dysfunction is an early event of atherosclerosis, and a few lncRNAs are involved in the regulation of EC function and structure (Table 3 and Figure 2) [113]. One such is the *MALAT1* lncRNA, known as the EC pro-inflammatory and angiogenesis lncRNA [114]. Expression of *MALAT1* is higher due to hyperglycemia, hypoxia, and oxidative stress [114]. Inhibition of *MALAT1* expression causes the reduction of S-phase cyclins (CCNB1, CCNB2, and CCNA2), as well as the increase in expression of *p21* and *p27Kip1* (cell cycle inhibitors), resulting in suppression of EC proliferation during hypoxia [114]. Like *MALAT1*, the expression of lncRNA *HOTTIP* increases in the coronary artery tissue of CAD patients and EC-stimulated with an inflammatory cytokine, TNF-α [115]. The increased expression of this lncRNA *HOTTIP* promotes EC proliferation and migration via the Wnt/β-catenin pathway [115]. Another study found that lncRNA *LINC00968* expression was higher in the coronary artery tissue of CAD patients. Upon stimulation of the oxidized low-density lipoprotein (oxLDL), this lncRNA expression was increased with EC proliferation and migration [116]. 

LncRNAs are also involved in recruiting leukocytes and macrophage-derived foam cells, although the evidence is limited (Table 3 and Figure 2). One such example is the lncRNA RP5-833A20.1 that is increased in foam cells [130]. Overexpression of this lncRNA in macrophages caused the increase in inflammatory cytokines (IL-1β, IL-6, and TNFα) by inducing miR-382-5p expression leading to the reduced expression of nuclear factor I A (*NFIA*) [130]. Similarly, another study found that lncRNA E330013P60 expression increased macrophages isolated from diabetic mice [121]. The increase in this lncRNA expression caused a shift to inflammatory phenotypes in those macrophages and foam cell formation [121]. 

The expressions of three lncRNAs, *GAS5*, *SNHG6*, and *ZFAS1*, were high in the atherosclerotic plaques [122]. Overexpression of GAS5 in macrophage-derived foam cells increased the intracellular lipid accumulation, whereas the inhibition of this lncRNA expression inhibited intracellular lipid accumulation and its progression atherosclerosis [123]. The action of *GAS5* lncRNA is mediated by its interaction with zeste homolog 2 (EZH2) to reduce the ATP-binding cassette transporter A1 (*ABCA1*) expression [123]. Interestingly, exosomes from the lncRNA *GAS5*-knockout macrophage cells prevented endothelial cell apoptosis upon the stimulation of oxidized LDL [155]. This evidence suggests that EC dysfunction could be prevented in the absence of *GAS5*. Another lncRNA associated with the atherosclerosis plaque is the MI-associated transcript (*MIAT*) that was upregulated in the serum samples of stroke patients with atherosclerosis, and in the mouse model of atherosclerosis [127,128]. LncRNA *MIAT* sponges the miR-181-b action to increase the *STAT3* expression thus promotes the proliferation of VSMC [128]. Similarly, the lncRNA Cholesterol Homeostasis Regulator of MiRNA Expression (*CHROME*) was also upregulated in plasma patients of CAD and inflammatory cells in the atherosclerosis plaque [120]. This lncRNA *CHROME* interacts with miR-27b, miR-33a, miR-33b and miR-128 to regulate cellular cholesterol homeostasis [120]. 

Previous GWAS studies to determine the genetic susceptibility in CAD found that lncRNA *ANRIL* was strongly associated with a greater risk of having CAD [40]. Expression of this lncRNA *ANRIL* was higher in atherosclerotic plaques and peripheral blood mononuclear cells from CAD patients [118]. LncRNA *ANRIL* binds to the components of the polycomb repression complex-1 (PRC-1) and -2 (PRC-2) [156,157], thus initiating the suppression of the INK4 locus to decrease the expression of p15 and p16 proteins. Both p15 and p16 proteins are the controllers of cell division that limit the cell lifespan, and loss of these proteins will allow for aberrant cell proliferation as seen in VSMC and EC [158]. Other lncRNAs that regulate and promote VSMCs proliferation and migration are the smooth muscle–induced lncRNA enhances replication (*SMILR*) [131], AK09865 [117], *H19* [124], *LINC00305* [125], *LNC*-*ANG362* [126], retinal ncRNA3 (*RNCR3*) [129], and *BANCR* [119]. 

Unlike the above lncRNAs with the adverse effects, the *MEG3* is a protective lncRNA for the heart. MEG3 lncRNA expression was lower in EC stimulated with high glucose in vitro and in vivo conditions [139]. Reduced expression of *MEG3* caused severe EC dysfunction, with evidence of microvascular leakage and inflammation, via the activation of the PI3K/Akt signaling pathway [139]. The mechanism of how *MEG3* lncRNA protects EC function is partially by sponging the miR-9 action to regulate the angiogenesis and proliferation processes [159]. Another protective lncRNA is the cardiac apoptosis-related lncRNA (*CARL*), which was highly expressed in myocardial EC and was reduced during the development of myocardial infarction (MI) and atherosclerosis [133]. *CARL* lncRNA acts as a miRNA sponge to miR-539 and thus inhibits the suppression of the *PHB2* gene, responsible for the regulation of mitochondrial function and apoptosis [134]. Overexpression of *CARL* caused a reduction of the *PHB2* and *BAX* expressions and increased anti-apoptotic *BCL2* [133] to prevent the cell apoptosis during MI. 

Furthermore, lncRNA *MANTIS* was also lower in EC from idiopathic pulmonary arterial hypertension (IPAH) patients and animal models [138]. Interestingly, lncRNA *MANTIS* interacts with the Brg1 complex. This Brg1 protein complex is activated following the cardiac stress during cardiac remodeling and regulates the key genes in angiogenesis genes such as *SOX18*, *SMAD6*, and *COUP*-*TFII* [138]. Other lncRNAs that prevents VSMC proliferation and migration are the LncRNA-RP11-714G18.1 [137], NEXN antisense RNA 1 (*NEXN-AS1*) [140], smooth muscle and endothelial cell-enriched migration/differentiation-associated long non-coding RNA (*SENCR*) [141,160], HOX transcript antisense RNA (*HOTAIR*) [135], *LINCRNA-P21* [136], and atherosclerotic plaque pathogenesis associated transcript (*APPAT*) [132]. Some of these lncRNAs have good diagnostic potential for CVD event prediction and will be discussed in the next section. 

### 4.2. LncRNA in Heart Development

As for cardiac development, Braveheart (BVHT) lncRNA was the first lncRNA found to be necessary for the activation of important cardiac genes and transcription factors for cardiogenic differentiation and development [161,162]. Following that, many studies have identified other lncRNAs such as *Fendrr* [163], *Linc1405* [164], *HoxBlinc* RNA [165], Handsdown (*Hdn*) [166] in mice, and (CAR)diac (M)esoderm (E)nhancer-associated (N)on-coding RNA (*CARMEN*) [167], Heart Brake LncRNA 1 (*HBL1*) [168] and *Ppp1r1b* [169] in human. The majority of these lncRNAs interact with Polycomb repressive complex-2 (PRC2), a histone methyltransferase responsible for epigenetic silencing during the development of the heart and body wall [170]. It is important to note that the exact mechanisms of how these lncRNAs regulate heart differentiation and development are not fully understood.

### 4.3. LncRNA in Cardiac Remodeling

Thickening of the ventricle wall and a reduction in the contractility of the heart are often seen in cardiac hypertrophy and remodeling, which could be fatal if the situation is prolonged. Among the lncRNAs that are involved in cardiac hypertrophy is the lncRNA cardiac hypertrophy-associated (*Chast*), which was increased in the mouse model of cardiac stress (transverse aortic constriction, TAC), as well as in human patients with aortic stenosis [145]. Overexpression of lncRNA *Chast* induced cardiac hypertrophy and remodeling in cardiomyocytes in vivo and in vitro models [145], whereas the silencing of this lncRNA prevented cardiac remodeling despite a stress-induction [145]. This cardiac hypertrophy action of *Chast* was shown due to its suppression of Pleckstrin homology domain-containing protein family M member 1 (*Plekhml*) expression that is responsible for cardiac autophagy [145]. Like *Chast*, the expression of cardiac-hypertrophy-associated epigenetic regulator (*Chaer*) lncRNA is increased during cardiac hypertrophy, and a heart-specific lncRNA [144]. Interestingly, a mouse model of lncRNA *Chaer* expression knockdown did not develop cardiac hypertrophy, with no substantial effect on the normal heart function and histology [144]. Therefore, this lncRNA *Chaer* is only involved in a pressure/stress-induced cardiac hypertrophy, and loss of its expression will not disrupt the normal heart function, hence this lncRNA is a good target for therapeutic strategy. 

Another lncRNA is the cardiac hypertrophy-related factor (*CHRF*), which was highly expressed in a cellular model of cardiac hypertrophy [146]. Following the angiotensin II treatment (Ang-II), the expression of lncRNA *CHRF* was higher and this lncRNA suppressed the miR-489 expression as its endogenous microRNA sponge [146]. The reduction of miR-489 expression caused an increase of the myeloid differentiation primary response gene 88 (*Myd88*), thus initiating the cardiac hypertrophy [146]. LncRNA BC088254 expression was increased in the rat model of cardiac hypertrophy [142]. In this study [142], the expression of BC088254 was negatively correlated with the *PHB2* gene, which is responsible for regulating mitochondrial function and apoptosis. Another reported lncRNA is the *lncRNA-ROR*, which was higher in cardiomyocytes of the TAC mouse model [147]. The mechanism of how *lncRNA-ROR* promotes cardiac hypertrophy is partially via its interaction with miR-133, in which they have an inverse relationship [147]. Loss of miR-133 expression led to the increase of cardiac myocytes and cardiac hypertrophy markers [171]. However, whether the inverse relationship between miR-133 and *lncRNA-ROR* expressions is due to microRNA sponge action or not is unknown. *MALAT1* lncRNA is a well-known pro-inflammatory lncRNA, and its expression was higher in the rat model of myocardial ischemia-reperfusion (I/R) injury [148]. Inhibition of *MALAT1* expression rescued the rats’ cardiac function due to a restoration of the PI3K/AKT signaling pathway [148]. Studies in the rat model of hypertension showed that *MALAT1* expression was increased in the heart [149,150]. Overexpression of *MALAT1* caused severe cardiac fibrosis and remodeling by reducing the expression of *MyoD* that is responsible for the contractile phenotype of the heart [149]. MALAT1 recruits the Suv39h1, a histone methyltransferase enzyme, to *MyoD* loci and causes H3K9me3 trimethylation to silence *MyoD* expression [149]. 

In terms of cardiac fibrosis, lncRNA WIsp2 SuPer-Enhancer associated RNA (*WISPER*) was reported to regulate fibrosis [153]. This lncRNA *WISPER* is a heart-enriched lncRNA, and its high expression was observed in MI-induced fibrosis of the mouse model and aortic stenosis patients [153]. Inhibition of this lncRNA expression prevented the MI-induced fibrosis and cardiac remodeling [153]. Besides that, in the mouse model of MI, Myocardial Infarction-Associated Transcript 1 (*MIRT1*) and 2 (*MIRT2*) expressions were significantly upregulated and correlated with the left ventricle remodeling [172]. However, the exact mechanism of how these lncRNAs promotes cardiac hypertrophy is unknown. In heart failure (HF) patients, antisense transcript of β-secretase-1 (*BACE1*), also known as lncRNA *BACE1-AS1*, was upregulated in left ventricle biopsies [143]. This high expression of *BACE-AS1* increased EC apoptosis and silencing its expression removed this effect [143]. Another study of HF patients showed that the expression of a non-coding repressor of NFAT (*NRON*) lncRNA was higher in plasma samples of HF patients [152]. 

As for protective lncRNA in cardiac remodeling, lncRNA *MHRT*, known as the myosin heavy-chain associated RNA transcript, is the main lncRNA with this function. This lncRNA *MHRT* is abundantly and specifically expressed in the heart [151]. In the mouse model of transverse aortic constriction (TAC) (to induce cardiac stress), the expression of lncRNA *MHRT* was low in cardiac tissues, and this inhibition was due to Brg1-Hdac-Parp chromatin repressor complex-3 that was activated following cardiac stress [151]. The suppression of *MHRT* expression allows for cardiac remodeling to occur as the Brg1 protein has a specific dual-binding helicase site that can be occupied by *MHRT* or its target genomic DNA region. Thus, restoration of *MHRT* expression prevented the Brg1 action by binding to this helicase domain and sequestering Brg1 to prevent cardiac remodeling [151]. This *MRHT*-Brg1 negative feedback action may explain how the heart responds to injury in the T2D environment.

In contrast, plasma expression of *MHRT* was increased in HF patients [152]. The discrepancies in the findings of *MHRT* expression may be due to different CVD models and sampling, as one study used the heart tissues while the latter used plasma samples. However, this difference may also due to the feedback regulatory function of lncRNA *MHRT* to protect the heart. A previous study of rat cardiomyocytes in an in vitro acute MI model showed that in response to oxidative stress, lncRNA *MHRT* expression was upregulated to reduce cell apoptosis and released into the circulation [173]. Considering its important function, lncRNA *MHRT* may serve as an excellent potential biomarker for MI and HF. Similar to *MHRT*, lncRNA *TINCR* expression was lower in the TAC mouse model [154]. This lncRNA prevented cardiac hypertrophy by binding to the PRC2 complex and recruiting it to the *CaMKII* gene promoter. Silencing of the *CaMKII* gene attenuated Ang-II-induced cardiomyocyte hypertrophy [154]. Identification of these lncRNAs in CVD showed that most of these lncRNAs are heart-specific and may be potentially used as biomarkers for CVD and heart dysfunction. 

## 5. Circulating LncRNA as Biomarkers for CVD

Multiple plasma proteins or peptides have been used as biomarkers to detect CVD events or predict their presence (Table 4). For example, to diagnose heart failure (HF), the measurement of brain natriuretic peptide (BNP) or N-terminal of prohormone BNP (NT-proBNP), ST-2, and atrial natriuretic peptide (ANP) are widely used [174]. For atherosclerosis or CAD, the level of troponin T (TnT), high sensitivity Troponin (HsTnT), and creatinine phosphokinase-MB are used [174,175]. However, the lack of early biomarkers to predict these CVD events (at least 1–2 years beforehand) is devastating, as early detection could help reduce the mortality risk. The presence of silent MI has a similar risk to the clinically diagnosed MI towards the HF incidence and death [176]. Therefore, prompting the urgent need to find more reliable biomarkers. One suggestion is to use the noncoding RNA biomarkers in the blood to improve the detection. A systematic review of circulating lncRNAs as a predictor of CVD future events showed that among the 30 studies, the lncRNA expression signatures have a moderate sensitivity with high specificity [177]. However, these lncRNAs have a strong potential to differentiate CVD patients from those without CVD or healthy controls (AUC: 0.85) [177].

Among those lncRNAs discussed in the previous section, some of them have been validated or investigated as potential biomarkers of HF for diagnostic purposes, especially in circulating blood or plasma samples. Examples are the increased expression of lncRNA *MIAT* [127,128], *CHROME* [120], *NRON*, and *MHRT* [152], and reduced expression of *APPAT* [132]. The first study to use lncRNA expression to predict CVD events is for detecting the left ventricular remodeling (LVM) after myocardial infarction (MI) in 788 patients [185]. In this study [185], a reduced expression of lncRNA uc022bqs.1 or *LIPCAR* was observed in the early stage of MI after the LVM, but its expression was increased in later stages as the condition worsened. Higher expression of *LIPCAR* was confirmed in the HF group with LVM, and even after the adjustment of other risk factors, the high expression of *LIPCAR* is an independent predictor of 3-year cardiovascular mortality (OR: 4.16) [185]. Importantly, for patients with the highest expression of *LIPCAR*, the mortality risk is even higher (OR: 32.58) [185]. Similarly, another study showed that expression of plasma *LIPCAR* correlated with the severity of coronary stenosis (AUC = 0.782) in the ST-segment elevation MI (STEMI) patients [186] and strongly predicts the CAD event (AUC: 0.722) [182]. 

In another study that involved HF patients, the levels of two lncRNAs, *NRON* and *MHRT*, in plasma samples could predict acute MI events with a sensitivity and specificity of 86.5% and 70.2%, respectively [152]. The expression of plasma *MHRT* was confirmed again in another study of chronic HF patients, in which the reduced expression of *MHRT* was associated with the worst outcomes of survival after the treatment [189]. A panel of nine lncRNAs (*CDKN2B-AS1*, *EGOT*, *H19*, *HOTAIR*, *LOC285194*, *RMRP*, *RNY5*, *SOX2-OT*, and *SRA1*) was able to differentiate the HF patients compared to controls [188]. In this study [188], *CDKN2B-AS1, H19, RMRP, RNY5, SOX2-OT* and *SRA1* were upregulated, whereas *EGOT, HOTAIR*, and *LOC285194* expressions were downregulated. High expression of lncRNA Heat2 was observed in peripheral blood mononuclear cells (PBMC) samples of HF patients, and this lncRNA expression is enriched in circulating immune cells [183]. Functional characterization of lncRNA *Heat2* showed that this lncRNA regulates immune cell division, invasion, transmigration, and adhesion to EC [183]. 

As for predicting acute MI (AMI) alone, peripheral blood mononuclear cells (PBMC) derived lncRNA *H19*, *MIAT*, and *MALAT1* were observed to be higher in AMI patients [181]. In this study, the diagnostic potential of lncRNA H19 to predict AMI was significant (AUC, 0.753; 95% CI, 0.689~0.817) and is associated with other CVD risk factors [181]. Another study showed that the plasma expression of lncRNA urothelial carcinoma-associated 1 (*UCA1*) was reduced in AMI 48 h after the event and started to increase afterward [190]. However, the diagnostic potential of this lncRNA *UCA1* to predict AMI is only minimal (AUC: 0.757) [190]. One study showed that the expression of three lncRNAs, the potassium voltage-gated channel, KQT-like subfamily, member 1 opposite strand/antisense transcript 1 (*KCNQ1OT1*), hypoxia-inducible factor 1A antisense RNA 2 (*HIF1A-AS2*) and *MALAT1* were increased in PBMC samples in MI patients, whereas lncRNA *ANRIL* expression was reduced [184]. Comparison between STEMI and non-STEMI patients showed that *ANRIL*, *KCNQ1OT1*, *MALAT1*, and *MIAT* expression were lowered in those with STEMI [184]. Interestingly, the expression of both *ANRIL* and *KCNQ1OT1* lncRNAs can correctly re-group the patients (left ventricular dysfunction) that was missed by the multiple clinical variables panel [184]. In contrast to the other lncRNAs, the expression of lncRNA *APPAT* was lower in blood samples of MI or angina pectoris patients with the potential to predict these CVD events at 78.72 % sensitivity and 93.02% specificity [132]. Similar to APPAT, lncRNA Zinc finger antisense 1 (*ZFAS1*) expression in plasma of AMI patients was reduced, however the diagnostic potential of this lncRNA to differentiate AMI from non-AMI patients, is minimal (AUC: 0.664) [179]. Interestingly, in this study [179], the expression of *ZFAS1* was opposite to another lncRNA, Cdr1 antisense (*CDR1AS*), in which its expression was increased in AMI patients with a similar diagnostic potential (AUC: 0.671). As for detecting hypertension or cardiac hypertrophy, expression of three lncRNAs, *MHRT*, *FENDRR* and *CARMEN* was higher in PBMC samples of hypertensive patients, though no diagnostic potential was measured in this study [191]. 

In ischaemic stroke (IS) patients, the plasma expression of lncRNA *MIAT* expression was higher and correlated with the level of high-sensitivity C-reactive protein, infarct volume, and poor prognosis [187]. Moreover, this lncRNA expression in plasma could differentiate IS patients from the controls (AUC: 0.842) [187]. Another study also confirmed that lncRNA *MIAT* expression was higher in serum samples of the stroke patient with evidence of atherosclerotic plaques [127]. 

For predicting CAD, one study found that the higher plasma expression of lncRNA AC100865.1 or known as the *CoroMarker*, predicted the CAD event in a cohort of 221 CAD patients and 187 controls, with a sensitivity of 78.05% and a specificity of 86.49% [180]. Importantly, high expression of *CoroMarker* lncRNA was only evident in CAD patients, not with other CVD or metabolic diseases [180]. Another study also discovered three lncRNAs in peripheral blood mononuclear cells (PBMC) samples of CAD patients, in which the *KCNQ1OT1*, *HIF1A-AS2*, and apolipoprotein A-1 antisense RNA (*APOA1-AS*) lncRNAs were higher in CAD patients [178]. The diagnostic potential of each lncRNAs (area under ROC curve) was 0.865 (*KCNQ1OT1*), 0.852 (*HIF1A-AS2*), and 0.967 (*APOA1-AS*). The combination of these lncRNAs in a panel predicted the CAD event with a better AUC value of 0.990, and these lncRNA expressions also correlated with the levels of NT-proBNP and HsTnT markers [178]. Expression of *H19* lncRNA was higher in the plasma of CAD patients, but this high expression of *H19* only minimally predicted the CAD event (AUC: 0.631) [182]. In contrast, the expression of lncRNA *GAS5* in the plasma samples of CAD patients was reduced and its diagnostic potential was shown to be high, with an AUC of 0.9783 [89], thus suggesting that *GAS5* lncRNA is a very promising biomarker for CAD. Although these lncRNAs have been extensively analyzed to predict CVD events, their roles in CVD complications of T2D patients still require clarifications. 

## 6. Circulating lncRNAs for CVD Complications in T2D

**Figure 3:**
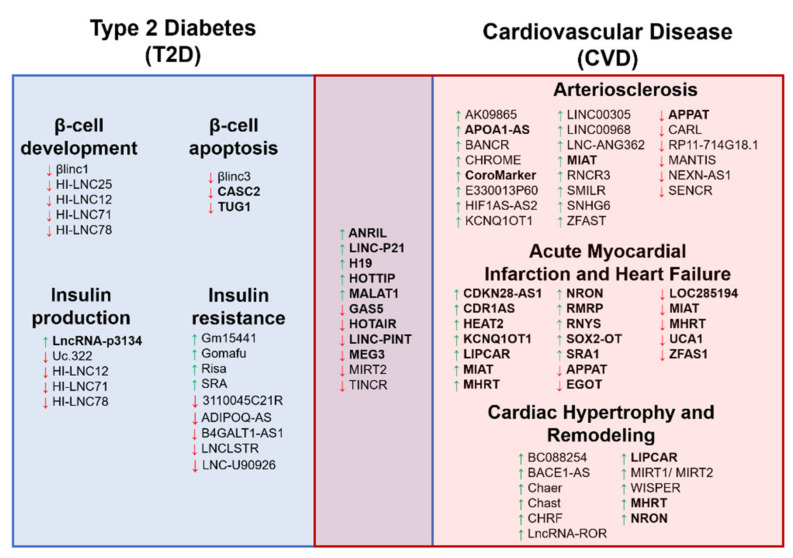
The schematic diagram illustrates the long noncoding RNA (lncRNA) involvement in both Type 2 Diabetes (T2D) and cardiovascular disease (CVD) complications. The diagram shows the overlapping of the lncRNAs reported in both T2D and CVD conditions. A green upward arrow indicates the upregulation of the lncRNA expression, a red downward arrow indicates the reduced expression, and the bold lncRNAs exist in the circulating biological samples for diagnostic potential.

Previous findings of lncRNAs associated with T2D or CVD showed that some of these lncRNAs are overlapped between the two disease conditions (Figure 3). Thus, previous studies determined the diagnostic potential of these lncRNAs as biomarkers for CVD complications in T2D. One such lncRNA is the *MALAT1*, in which its expression was higher when cardiomyopathy occurred in diabetic rats [192]. A study of T2D patients showed that *LINC-PINT* expression in the plasma was lower in T2D patients. However, this reduction worsens when these T2D patients developed cardiomyopathy in the six-years follow-up visit [77]. Another is the lncRNA *ANRIL* expression, which was reported higher in T2D patients, and circulating *ANRIL* expression becomes much higher in T2D patients with MI, thus indicating the potential of *ANRIL* in detecting the CVD complication in T2D patients [92]. In another study of well-controlled diabetic patients, plasma expression of lncRNA *LIPCAR* correlated with left ventricular grade I diastolic dysfunction. The expressions of *MIAT* and *SENCR* lncRNAs correlated with the left ventricular remodeling in these diabetic patients [193]. As for other lncRNAs in Figure 3, these lncRNAs were reported separately in T2D and CVD conditions, and thus require validation in T2D subjects that developed CVD complications compared with those who did not. Despite being limited, this evidence suggests that circulating lncRNA expression could be used as the potential biomarkers to predict the early events of diabetic cardiomyopathy. Further identification of candidate lncRNAs is important as the T2D environment itself is a risk factor for CVD complications and may elevate the biomarkers’ levels more or differently. Nevertheless, these findings are still limited; thus, the interpretations of the results require more studies to confirm the conclusion.

## 7. Conclusions

Despite the availability of various treatments for CVD and T2D, the mortality risk of T2D patients due to CVD remains high. Thus, identifying high-risk individuals among T2D patients is crucial for better disease management. One way of doing so is using the circulating long noncoding RNAs to complement the clinical variables to develop a better risk and prediction scoring system. Among the studies of lncRNAs as biomarkers, the most promising lncRNAs are from MI and HF of T2D studies, as the findings are more consistent and showed strong predictive values. Notably, most of these lncRNAs are heart-specific and exist in the circulating biological samples, making them potential biomarkers for future CVD events. Expressions of several lncRNAs (*ANRIL*, *LINC-PINT*, *MALAT-1*, *LIPCAR*, *SENCR*, and *MIAT*) have been measured in T2D individuals with CVD complications and could predict these CVD events. Although lncRNAs such as *GAS5, H19, HOTAIR, HOTTIP, LINC-P21, MEG3, MIRT2*, and *TINCR*, are reported in T2D and CVD individuals separately. Thus, further validation and confirmation of these lncRNAs in T2D individuals with CVD progression are needed. With more lncRNAs being identified and the growing understanding of the molecular mechanisms and roles of these lncRNAs, the application of using these lncRNAs as new biomarkers will allow for early identification of CVD complications in high-risk T2D patients. 

## Figures and Tables

**Table 1:** Summary of the reported long non-coding RNAs (lncRNAs) in Type 2 Diabetes (T2D). List of previously published lncRNAs in T2D environment and their respective information.

LncRNA	Expression in T2D	Sample Types	Origin	Function	Reference
Insulin secretion					
*KCNQ1OT1*	High	Islets	Healthy controls and T2D patients	Unknown but is located in the region near to T2D loci	[41]
*LINC-P21*	High	Serum, islets	Healthy controls and T2D patients, INS-1 cells	Reduces β-cell insulin secretion	[42]
*LncRNA-p3134*	High	Serum exosomes	Healthy controls and T2D patients	Involves in β-cell insulin secretion	[43]
*βlinc1*	Low	Islets	βlinc1^-/-^ mice, MIN6 cells	Important for islet development	[44]
*βlinc3*	Low	Islets	C57BL/6J mice, MIN6 cells, Healthy controls and T2D patients	Involves in β-cell apoptosis	[45]
*GAS5*	Low	Islets	Db/db (diabetes) mice, MIN6 cells	Regulates insulin synthesis and secretion	[46]
	Low	Serum, islets	Healthy controls and T2D patients, INS-1 cells	Regulates insulin secretion	[47]
*H19*	Low	Serum	Healthy controls and T2D patients	Correlated with hypertension and weight lost	[48]
*HI-LNC25*	Low	Islets	Healthy controls and T2D patients	Involves in β-cell development	[41]
*HI-LNC12*	Low	Islets	EndoC-βH1 cells	Involves in β-cell development and insulin secretion	[49]
*HI-LNC45*	Low	Islets	Healthy controls and T2D patients	Unknown but is located in the region near to T2D loci	[41]
*HI-LNC71/PLUTO*	Low	Islets	EndoC-βH1 cells	Involves in β-cell development and insulin secretion	[49]
*HI-LNC78*	Low	Islets	EndoC-βH1 cells	Involves in β-cell development and insulin secretion	[49]
*HOTTIP*	Low	Islets	Db/db mice, MIN6 cells	Regulates insulin secretion and β-cell cycle	[50]
*LINC-PINT*	Low	Islets	Db/db mice, MIN6 cells	Regulates insulin synthesis and secretion	[51]
*MEG3*	Low	Islets	Healthy controls and T2D patients	Involves in β-cell apoptosis	[52]
	Low	Islets	Db/db mice, MIN6 cells	Involves in β-cell insulin production and apoptosis	[53]
*TUG1*	Low	Islet	NOD mice, MIN6 and Nit-1 cells	Involves in β-cell apoptosis and promotes insulin secretion	[54]
Uc.322	Low	Islets	C57BL/6J mice, MIN6 cells	Promotes β-cell insulin secretion	[55]
*Insulin resistance*					
*Gomafu*	High	Liver	High-fat diet and ob/ob mice	Promotes insulin resistance	[56]
*HOTAIR*	High	Liver	Healthy controls and T2D patients	Promotes insulin resistance	[57]
*MALAT1*	High	Liver	High-fat diet and ob/ob mice	Promotes insulin resistance	[58]
*MEG3*	High	Liver	High-fat diet and ob/ob mice	Promotes insulin resistance	[59]
*Risa*	High	Liver	Insulin resistance cell model	Promotes insulin resistance	[60]
*SRA*	High	Liver	Knockout mice and cell line	Promotes lipid accumulation	[61]
*B4GALT1-AS1/LNCSHGL*	Low	Liver	High-fat diet and Db/db mice	Prevents insulin resistance	[62]
*LNCLSTR*	Low	Liver	Knockout mice, ApoE-/-mice	Regulates triglycerides level	[63]
*MIRT2*	Low	Liver	Db/db and ob/ob mice	Prevents insulin resistance	[64]
Gm15441	High	Skeletal muscle	Db/db mice	Involves in insulin sensitivity	[65]
*Risa*	High	Skeletal muscle	Insulin resistance cell model	Promotes insulin resistance	[60]
*H19*	Low	Skeletal muscle	Db/db mice	Prevents insulin resistance	[66]
	Low	Skeletal muscle	High-fat diet mice	Promotes insulin sensitivity	[67]
3110045C21Rik	Low	Skeletal muscle	Db/db mice	Involves in insulin sensitivity	[65]
*TINCR*	High	Adipocyte	3T3-L1 insulin resistance cell model	Promotes inflammation	[68]
*ADIPOQ-AS*	Low	Adipocyte	High-fat diet mice	Prevents insulin resistance	[69]
*LNC-U90926*	Low	Adipocyte	Db/db and ob/ob mice	Prevents insulin resistance	[70]

**Table 2:** Summary of the reported circulating long non-coding RNAs (lncRNAs) as biomarkers for Type 2 diabetes (T2D). List of previously published circulating lncRNAs as biomarkers for T2D and their respective information.

LncRNA	Expression in T2D	Sample Types	Origin	Function	Reference
*ANRIL*	High	PBMC	Healthy patients and T2D patients with or without myocardial infarction (MI)	Increases the risk of having T2D and MI	[92]
ENST00000550337.1	High	Blood	Healthy controls and T2D patients	Can differentiate between T2D patients and healthy controls	[84]
TCONS_00007244	High	Blood	Healthy controls and T2D patients	Can differentiate between T2D patients and healthy controls	[84]
TCONS_00000886	High	Blood	Healthy controls and T2D patients	Can differentiate between T2D patients and healthy controls	[84]
*CASC2*	Low	Serum	Healthy controls and T2D patients	Can differentiate between T2D patients and healthy controls	[88]
*CTBP1-AS2*	Low	PBMC	Healthy controls and T2D patients	Can differentiate between T2D patients and healthy controls	[85]
*GAS5*	Low	Serum	Healthy controls and T2D patients	Increases the risk of having T2D	[82]
	Low	Serum	Healthy controls and T2D patients	Increases the risk of having T2D	[89]
*HCG27_201*	Low	PBMC	Healthy controls and T2D patients	Can differentiate between T2D patients and healthy controls	[86]
*LINC00523*	Low	PBMC	Healthy controls and T2D patients	Can differentiate between T2D patients and healthy controls	[87]
*LINC00994*	Low	PBMC	Healthy controls and T2D patients	Can differentiate between T2D patients and healthy controls	[87]
*LINC-PINT*	Low	Plasma	Healthy controls and T2D patients	Can differentiate between T2D patients and healthy controls	[77]
*LY86-AS1*	Low	PBMC	Healthy controls and T2D patients	Can differentiate between T2D patients and healthy controls	[86]
*VIM-AS1*	Low	PBMC	Healthy controls and T2D patients	Can differentiate between T2D patients and healthy controls	[85]

Abbreviation: MI, Myocardial infarction; PBMC, Peripheral blood mononuclear cells; T2D, Type 2 diabetes.

**Table 3:** Summary of the reported long noncoding RNAs (lncRNAs) in cardiovascular disease (CVD). List of previously published lncRNAs in cardiovascular diseases and their information.

LncRNA	Expression in Disease	Cell Types	Origin	Function	Reference
Arteriosclerosis					
AK09865	High	VSMC	Hypertensive patients	Promotes VSMC cell proliferation	[117]
*ANRIL*	High	Atherosclerotic plaques, PBMC	CAD patients	Suppresses INK4 locus for aberrant cell proliferation	[118]
*BANCR*	High	Atherosclerotic plaques	CAD patients	Promotes VSMC proliferation	[119]
*CHROME*	High	Plasma, Inflammatory cells	CAD patients	Regulates cellular cholesterol homeostasis	[120]
E330013P60	High	Macrophages-derived foam cells	Diabetic db/db mice	Promotes inflammation and foam cell formation	[121]
*GAS5*	High	Atherosclerotic plaques	Arteriosclerosis patients and rats	Associates with arteriosclerosis plaque	[122]
	High	Macrophages-derived foam cells	Arteriosclerosis patients and rats	Promotes lipid accumulation Suppresses *ABCA1* expression via EZH2	[123]
*H19*	High	VSMC, atherosclerotic plaques	Stroke patients	Promotes VSMC cell proliferation Increases atherosclerotic plaques size	[124]
*HOTTIP*	High	Arterial tissue	CAD patients	Promotes EC proliferation	[115]
*LINC00305*	High	Atherosclerotic plaques ASMC cells	Arteriosclerosis patients	Promotes inflammation	[125]
*LINC00968*	High	Arterial tissue	CAD patients	Promotes EC proliferation	[116]
*LNC-ANG362*	High	VSMC	Cell line and rats treated with Ang II	Promotes VSMC cell proliferation	[126]
*MALAT-1*	High	EC	Hypoxia treated EC	Promotes EC proliferation and switching	[114]
*MIAT*	High	Serum	Stroke patient with atherosclerotic plaques, ApoE^−/−^ mice fed a high-fat diet	Promotes VSMC proliferation	[127,128]
*RNCR3*	High	Atherosclerotic plaques	Arteriosclerosis patients and mice	Promotes EC dysfunction and VSMC proliferation	[129]
RP5-833A20.1	High	Macrophages-derived foam cells	Macrophage–derived foam cells treated with oxidized LDL	Promotes inflammatory cytokines production	[130]
*SMILR*	High	Primary human saphenous vein–derived endothelial cells	CAD patients	Promotes VSMC cell proliferation	[131]
*SNHG6*	High	Atherosclerotic plaques	Arteriosclerosis patients and rats	Associates with arteriosclerosis plaque	[122]
*ZFAS1*	High	Atherosclerotic plaques	Arteriosclerosis patients and rats	Associates with arteriosclerosis plaque	[122]
*APPAT*	Low	VSMC, Blood	Patients with angina pectoris or MI	Maintains VSMC phenotypes	[132]
*CARL*	Low	Myocardial endothelial cells	Rat model of arteriosclerosis and MI	Prevents cell apoptosis Suppresses miR-539 expression Suppresses *PHB2* and *BAX* expression Increases anti-apoptotic protein *BCL2* expression	[133,134]
*HOTAIR*	Low	Atherosclerotic plaques	CAD patients	Regulates EC proliferation and migration	[135]
*LINCRNA-P21*	Low	VSMC	Cell model of arteriosclerosis, ApoE^-/-^ mice fed with high fat diets	Prevents VSMC cell apoptosis	[136]
RP11-714G18.1	Low	Atherosclerotic plaques VSMC	CAD Cell model of arteriosclerosis	Prevents VSMC cell migration, adhesion of EC to monocytes	[137]
*MANTIS*	Low	EC	Idiopathic pulmonary arterial hypertension patients, and rats	Regulates angiogenesis	[138]
*MEG3*	Low	EC	STZ-induced diabetic mice, EC cells	Regulates EC function via PI3K/Akt signaling	[139]
*NEXN-AS1*	Low	Atherosclerotic plaques	CAD, AMI or HF patients	Prevents adhesion of EC to monocytes	[140]
*SENCR*	Low	Vascular tissue	Premature CAD patients	Promotes EC proliferation	[141]
*Cardiac Remodeling*					
BC088254	High	Heart tissue	Rat model of TAC	Promotes cardiac hypertroph	[142]
*BACE1-AS*	High	Left ventricle biopsies	HF patients	Promotes EC apoptosis	[143]
*Chaer*	High	Heart tissue	Mouse model of TAC, Aortic stenosis patients	Promotes cardiac hypertrophy	[144]
*Chast*	High	Heart tissue	Mouse model of TAC, Aortic stenosis patients	Promotes cardiac hypertrophy Suppreses *Plekhml* expression and cardiac autophagy	[145]
*CHRF*	High	Heart tissue	Cardiomyocytes with Ang-II treatment	Promotes cardiac hypertrophy Suppresses miR-489 expression Increases *Myd88* expression	[146]
*LNCRNA-ROR*	High	Heart tissue	Rat model of TAC	Promotes cardiac hypertrophy	[147]
*MALAT1*	High	Heart tissue	Rat model of myocardial ischemia-reperfusion (I/R) injury	Promotes inflammation Suppresses PI3K/AKT pathway	[148]
	High	Heart tissue	Rat model of hypertension	Promotes cardiac fibrosis Suppresses *MyoD* expression	[149,150]
*MIRT1/MIRT2*	High	Heart tissue	Mouse model of MI	Correlates with left ventricle remodeling	[151]
*MHRT*	High	Plasma	HF patients	Predicts HF event	[152]
*WISPER*	High	Heart tissue	Mouse model of MI Aortic stenosis patients	Promotes cardiac fibrosis	[153]
*MHRT*	Low	Heart ventricles tissue	Mouse model of TAC	Cardio-protective lncRNA Suppresses BRG1-induced chromatin remodeling	[151]
*NRON*	High	Plasma	HF patients	Predicts HF event	[152]
*TINCR*	Low	Heart tissue	Mouse model of TAC	Cardio-protective lncRNA Suppresses *CaMKII* expression to regulate the ion channels	[154]

Abbreviation: AMI, Acute myocardial infarction; Ang-II, angiotensin II; ASMC, Aortic smooth muscle cells; CAD, Coronary artery disease; CVD, Cardiovascular disease; EC, Endothelial cell; HF, Heart failure; LDL, Low-density lipoproteins; MI, myocardial infarction; PBMC, Peripheral blood mononuclear cells; TAC, Transverse aortic constriction; VSMC, Vascular smooth muscle cells.

**Table 4:** Summary of the reported circulating long noncoding RNAs (lncRNAs) as biomarkers for cardiovascular disease. List of previously published circulating lncRNAs in cardiovascular diseases and their information.

LncRNA	Expression in Disease	Cell Types	Origin	Function	Reference
*APOA1-AS*	High	PBMC	CAD patients	Predicts CAD event	[178]
*CDR1AS*	High	Blood	AMI patients	Predicts AMI event	[179]
*CHROME*	High	Plasma, Inflammatory cells	CAD patients	Regulates cellular cholesterol homeostasis	[120]
*CoroMarker*	High	Plasma	CAD patients	Predicts CAD event	[180]
*H19*	High	PBMC	AMI patients	Predicts AMI event	[181]
	High	Plasma	CAD patients	Predicts CAD event	[182]
*HEAT2*	High	PBMC	HF patients	Predicts HF event	[183]
*HIF1AS-AS2*	High	PBMC	CAD patients	Predicts CAD event	[178]
	High	PBMC	AMI patients	Predicts AMI event	[184]
*KCNQ1OT1*	High	PBMC	CAD patients	Predicts CAD event	[178]
	High	PBMC	AMI patients	Predicts AMI event	[184]
*LIPCAR*	High	Plasma	HF patients	Predicts CVD death after HF	[185]
	High	Plasma	CAD patients	Predicts CAD event	[182]
	High	Plasma	AMI patients	Predicts ST-segment elevation MI (STEMI) event	[186]
*MALAT1*	High	PBMC	AMI patients	Predicts AMI event	[181]
	High	PBMC	AMI patients	Predicts AMI event	[184]
*MIAT*	High	Plasma	Ischaemic stroke patients	Can differentiate stroke patients from controls	[187]
	High	PBMC	AMI patients	Predicts AMI event	[181]
*MHRT*	High	Plasma	HF patients	Predicts HF event	[152]
*NRON*	High	Plasma	HF patients	Predicts HF event	[152]
*A panel of 9 lncRNAs*					
*CDKN2B-AS1*	High	PBMC, heart tissue	HF patients	Predicts ischemic HF event	[188]
*RMRP*	High				
*RNY5*	High				
*SOX2-OT*	High				
*SRA1*	High				
*EGOT*	Low				
*HOTAIR*	Low				
*LOC285194*	Low				
*APPAT*	Low	VSMC, Blood	Patients with angina pectoris or MI	Maintains VSMC phenotypes	[132]
*ANRIL*	Low	PBMC	AMI patients	Predicts acute MI event	[184]
*GAS5*	Low	Plasma	CAD patients	Predicts CAD event	[89]
*MIAT*	Low	PBMC	AMI patients	Predicts AMI event	[184]
*MHRT*	Low	Plasma	Chronic HF patients	Predicts survival after treatment	[189]
*UCA1*	Low	Plasma	AMI patients	Predicts acute MI event	[190]
*ZFAS1*	Low	Blood samples	AMI patients	Predicts acute MI event	[179]

Abbreviation: AMI, Acute myocardial infarction; Coronary artery disease; CVD, Cardiovascular disease; HF, Heart failure; MI, myocardial infarction; PBMC, Peripheral blood mononuclear cells; VSMC, Vascular smooth muscle cells.

## Data Availability

Not applicable.
